# Sequelae and Animal Modeling of Encephalitic Alphavirus Infections

**DOI:** 10.3390/v15020382

**Published:** 2023-01-28

**Authors:** Rachel A. Reyna, Scott C. Weaver

**Affiliations:** 1 Department of Pathology, University of Texas Medical Branch, Galveston, TX 77555, USA; 2 Department of Microbiology and Immunology, University of Texas Medical Branch, Galveston, TX 77555, USA; 3 Institute for Human Infections and Immunity, University of Texas Medical Branch, Galveston, TX 77555, USA

**Keywords:** alphavirus, animal models, encephalitis, sequelae

## Abstract

Eastern (EEEV), Venezuelan (VEEV), and western equine encephalitis viruses (WEEV) are members of the genus *Alphavirus*, family *Togaviridae*. Typically spread by mosquitoes, EEEV, VEEV, and WEEV induce febrile illness that may develop into more severe encephalitic disease, resulting in myriad severe neurologic sequelae for which there are no vaccines or therapeutics. Here, we summarize the clinical neurologic findings and sequelae induced by these three encephalitic viruses and describe the various animal models available to study them. We emphasize the crucial need for the development of advanced animal modeling combined with the use of telemetry, behavioral testing, and neuroimaging to facilitate a detailed mechanistic understanding of these encephalitic signs and sequelae. Through the use of these systems, much-needed therapeutics and vaccines can be developed.

## 1. Introduction

Alphaviruses are members of the *Togaviridae* family [1]. These viruses are spherical and enveloped, with single-stranded, positive-sense RNA genomes [2]. The 9.7–11.8 kilobase (kb) genome encodes a total of ten proteins: four nonstructural proteins (nsP1 through nsP4) and six structural proteins (E1, E2, E3, capsid, transframe [TF], and 6 K) [3,4]. The four nonstructural proteins each play a role in viral replication, while the structural proteins are crucial for viral assembly [4].

There are over 30 identified species of alphaviruses [1]. Members of the genus *Alphavirus* can be grouped both antigenically and geographically into eight antigenic/genetic complexes [2,5]. Geographically, alphaviruses are separated into Old World and New World viruses and are present on every continent except Antarctica [2,5,6]. Old World viruses are found throughout Asia, Africa, and Europe [7]. Human pathogenic Old World alphaviruses include Barmah Forest (BFV), chikungunya (CHIKV), o’nyong-nyong (ONNV), Ross River (RRV), and Sindbis viruses (SINV) [5,7]. New World alphaviruses occur throughout the Americas [7], and eastern equine encephalitis (EEEV), Mayaro (MAYV), western equine encephalitis (WEEV), Una (UNAV), and Venezuelan equine encephalitis viruses (VEEV, and related viruses in the VEE complex) cause disease in humans [5,7]. 

Most alphaviruses are transmitted by hematophagous arthropods [4,8]. While the majority are spread by mosquitoes, lice, ticks, and cliff swallow bugs may also play a role in transmission [6,8,9,10]. Due to the wide range of hosts targeted by these hematophagous vectors, alphaviruses are maintained within a variety of susceptible vertebrate hosts that generate sufficient viremia to propagate the transmission cycles [4]. As there are no approved vaccines against alphaviral diseases, the best forms of protection include basic prevention against mosquito contact: using insect repellant, wearing clothing to cover arms and legs, using mosquito nets, and eliminating nearby aquatic larval habitats [11]. 

The objective of this review is to briefly summarize the clinical findings and sequelae of the three main encephalitic alphaviruses causing severe disease and sequelae in humans: EEEV, VEEV, and WEEV. Current animal models will also be reviewed, with a focus on their manifestations of various sequelae. Complete understanding of the mechanisms driving pathogenesis and sequelae for these encephalitic viruses is crucial for the development of vaccines and therapeutics. Examining where the deficiencies and advantages lie within the available animal models can inform the development of models that will accurately portray the diseases they study. In doing so, therapeutics can be developed for those suffering these debilitating sequelae. 

## 2. New World Encephalitic Alphaviruses

New World alphaviruses are known to induce encephalitic sequelae. The three predominant viruses in this category include EEEV, VEEV, and WEEV [8]. There are no approved vaccines to protect against disease caused by these viruses [12,13]. However, there are formalin-inactivated and live-attenuated vaccines provided to laboratory workers as well as members of the United States military, such as the live-attenuated VEEV strain TC-83 [14]. Up to half of TC-83 vaccinees suffer adverse events, including fever, headache, malaise, myalgia, respiratory symptoms, sore throat, loss of appetite, transient electrocardiographic abnormalities, and even spontaneous abortion, and many do not seroconvert [15,16,17]. All signs and symptoms resolve within 36 h [15,16]. Treatment for those infected with New World alphaviruses is limited to supportive care [13,14]. Due to their high lethality, incapacitating disease, and aerosolization potential, these agents have historically been investigated for their use as biological weapons [18].

### 2.1. Eastern Equine Encephalitis Virus (EEEV)

EEEV occurs predominately throughout eastern North America as well as periodically in the Caribbean [8,19]. Variants found in North America tend to be more virulent than strains found in Central or South America [19]. These marked differences in location and severity led to the South American strains of EEEV being reclassified as their own virus and species, Madariaga virus (MADV) [20]. 

Few cases of EEE are reported each year, an average of 11, and epidemiologic investigations suggest that only around 4–5% of infections result in reported disease [21,22]. The incubation time ranges from 4 to 10 days and infection results in febrile illness with or without neurologic signs [12]. An estimated one-third of diagnosed cases are fatal, although this can be elevated to up to 90% [8,12,22,23]. Patients that develop no signs of neurologic involvement during the acute phase tend to recover completely, with no lasting sequelae [8,12]. Less than 5% of epidemiologically suggested cases develop signs or symptoms of neurologic disease, which include vomiting, seizures, diarrhea, fever, headache, coma, drowsiness, and behavioral changes [12,22,24,25]. The onset is often dependent on age, with younger patients developing signs earlier during acute disease as compared to older children and adults [12]. Age also plays a role in the severity of disease, with patients under 15 and over 50 being more likely to develop severe illness [21]. Another factor is the length of the prodromal phase prior to development of neurologic signs, with a longer prodrome being correlated with a more favorable outcome [26]. 

At least half or as high as 90% of neurologic cases develop lasting sequelae [14,24,27], including seizures, convulsions, altered mental status, somnolence, and intellectual disability [14,26,28]. This results in a very high cost of typically institutionalized care, often for life [29]. Follow-up evaluations of survivors indicate a potential for cognitive improvement and motor rehabilitation; 40% of patients overall demonstrate improvement, whereas 75% of those with severe deficits demonstrate sequelae improvement [26]. The exact cause of these sequelae remains unknown. Autopsy findings indicate viral-induced inflammatory and necrotic changes correlated to central nervous system (CNS) regions with abnormalities observed by magnetic resonance imaging (MRI) [25,26]. MRI scan results obtained without contrast may demonstrate gradually increasingly obvious foci of fluid-attenuated inversion recovery (FLAIR) and T2 abnormalities within the basal ganglia, thalamus, and cortex, ultimately indicating diffuse encephalitis [25,30,31]. Computed tomography (CT) scans are not typically helpful in diagnosis and show no to minimal pathologic changes [25,30,31]. 

Animal modeling of EEE in mice, guinea pigs, and macaques suggests that viral entry into the brain, inducing the associated neurologic disease and sequelae, may occur through the olfactory bulb in the case of aerosol exposure [23,27,32]. However, the related VEE and WEE viruses can enter murine brains through the circumventricular organs before spreading to other regions after peripheral infection; this non-olfactory entry route cannot be ruled out for EEEV following peripheral infection [33]. Moreover, the cause of neurologic signs may be from neurologic dysfunction as opposed to neuronal death, as suggested by the lack of histopathological lesions within the brain [27]. As EEEV has previously been studied as a potential bioweapon, understanding all potential mechanisms of neurologic infiltration and disease following respiratory infection is crucial for vaccine and therapeutic development. 

However, due to the small number of annual cases and typically high mortality rate, further clinical characterization of EEEV-induced sequelae is difficult. The development of therapies to minimize sequelae and promote recovery relies on animal modeling. Current lethal models resulting in neurologic signs include mice, golden hamsters, guinea pigs, and non-human primates including cynomolgus macaques and the common marmoset (see Table 1) [23,27,32,34,35,36,37,38,39,40,41,42]. The majority of modeling is focused on intranasal or aerosol exposure, which may be relevant from a biodefense perspective [32,34,35,37,38,40,41]. However, as natural infections occur via mosquito transmission, models that characterize pathogenesis through subcutaneous exposure are also important [36,37,39,42].

BALB/c mice intranasally inoculated with EEEV develop encephalitis and neurologic signs just prior to death, including tremors and altered behavior, as indicated by a fixed gaze, inability to respond to stimuli, and reduced motor control [34]. C57BL/6 mice inoculated via the footpad develop neurologic signs including tremors [39]. Golden Syrian hamsters develop coma and stupor, with histopathological analysis indicating encephalitis and vasculitis within the brain [36]. While guinea pigs are less susceptible to disease than mice, they develop neurologic signs at a higher rate, including encephalitis and coma [32]. Macaques infected via aerosol develop neurologic signs including tremors, seizures, twitching, ataxia, head pressing, and nystagmus [38,41]. Cutaneous infection of macaques results in ataxia, tremors, lethargy, and imbalance [42]. Marmosets infected via aerosol develop neurologic signs including tremors, behavioral changes, and imbalance [35,40]. While all of these models develop visible neurologic signs that are valuable for the development of vaccines and therapeutics, researchers are limited to findings typically observed during general health checks. These findings are complicated by the effects of human presence in the vivarium, which likely interferes with observations of many neurologic signs, especially when working with non-human primates.

Advancements in the application of telemetry and remote monitoring have given researchers great insight into the development of sequelae using a cynomolgus macaque model for EEE [23,38,41,43]. Data from these studies provide valuable information that is inaccessible by simple daily observations, even by highly trained veterinarians, and periodic body weight/temperature measurements. Implanted telemetric devices indicate abnormalities on electroencephalographs (EEG) in regions associated with deep sleep as well as increased intracranial pressure [38,41]. Another telemetric study examined the changes in electrocardiography (ECG) with alterations correlated with fever [43].

Recently, more advanced telemetric devices have been implanted into macaques prior to infection, allowing researchers to conduct constant monitoring of EEG, ECG, heart rate, activity, body temperature, respiration rate, and blood pressure [23]. These primates were then exposed to EEEV strain V105 through aerosol challenge and monitored continuously via telemetry and staff observations through 140 h post-infection. Data from the telemetric devices were time-matched to baseline activity and split into daytime and nighttime windows. Overall, dramatic changes were detected, and activity measurements indicated disruption of the circadian rhythm, decreased consumption of food and fluid, as well as seizures presenting within the last few hours of the study [23].

### 2.2. Venezuelan Equine Encephalitis Virus (VEEV)

Venezuelan equine encephalitis virus (VEEV) occurs from Mexico through northern South America [19,44], and causes outbreaks in both equids and humans, resulting in a potential for great impact on the agricultural economy of endemic regions [19]. The VEE complex consists of various antigenic subtypes, with each having unique characteristics as pertain to epidemiology, ecology, and virulence [19,44]. For example, subtypes IAC and IC cause severe disease in both humans and equids, while subtype ID only causes disease in humans [19].

The majority of VEE cases are symptomatic but present only with signs of nonspecific febrile illness, which occur after a short incubation time ranging from 27.5 h to four days post-exposure [19,45]. Signs and symptoms include fever, chills, headache, myalgia, retro-orbital pain, nausea, vomiting, and arthralgia [44,45,46,47]. These are very similar to those caused by dengue virus, a flavivirus common in endemic regions, often resulting in misdiagnosis and underestimation of the VEE burden [44,48]. This results in the underestimation of case numbers, severity, and the economic impact of VEE.

Neurologic signs appear in an estimated 14% of cases, with case fatality rates ranging from 1 to 10% [14,19,49]. These signs include drowsiness, coma, seizures, convulsions, depression, disorientation, paralysis, and encephalitis [19,44]. The onset of neurologic signs appears to be age related; 35% of cases under 5 years of age develop neurologic signs as opposed to 5% of cases under 15 [19,44,49]. Up to 14% of survivors suffer myriad neurologic sequelae, including paralysis, convulsions, somnolence, seizures, coma, confusion, epilepsy, intellectual disability, altered personality, mental incapacity, photophobia, hearing loss, anosmia, ageusia, depression, recurrent headaches, and fatigue [14,19,44,45,50].

Due to the high rates of misdiagnosis and a paucity of autopsy and other clinical samples, full characterization of clinical VEE is difficult. Current data indicate that VEEV entry to the murine brain after subcutaneous exposure may occur in areas lacking a blood–brain barrier, including the hypothalamus, pineal body, area postrema, and the anteroventral third ventricle region [33]. Other research indicates that neuroinvasion following subcutaneous infection may occur after viral replication within the olfactory and dental tissues [51]. As with EEEV, studies examining VEEV infection both via subcutaneous and aerosol or intranasal exposure are important to better understand both natural and deliberate routes of exposure. Reliable animal models are crucial for the development of effective vaccines and therapeutics, as well as for understanding mechanisms driving neurologic signs and sequelae. Table 2 outlines the neurologic signs of clinical infection in comparison to current animal models.

BALB/c mice receiving either aerosol or subcutaneous inoculation develop lethal illness, succumbing to infection before the neurologic phase of disease [52,53,54]. CD-1 mice have previously been used to study VEEV neuroinvasion via subcutaneous footpad injection, which is lethal, with mice developing hind limb paralysis [33,51,55]. CB17 mice develop a lethal infection following subcutaneous exposure, with all exhibiting paralysis and histological encephalitis prior to death [56]. Severe combined immunodeficiency (SCID) mice also develop severe neurologic signs post-subcutaneous exposure; all develop behavioral changes leading to severe aggression, followed by ataxia and an unresponsiveness to stimuli before succumbing to infection [56]. No encephalitis or paralysis is observed in SCID mice [56]. Golden Syrian hamsters develop lethal disease post-aerosol exposure [58]. Subcutaneous infection also results in lethal disease without signs of encephalitis, although severe brain lesions are present upon histological exam [57]. Secondary bacterial infections may contribute to fatality, as indicated by lesions within the intestinal membranes [57]. Guinea pig models are less clinically relevant and are typically used to model equine infections with strain-specific lethality [62,63].

VEEV infection is non-lethal in the cynomolgus macaque model and rarely results in neurologic signs or sequelae, consistent with most human infections [61]. Cutaneous infection yields a trend of weight loss; transient viremia is detected through 3 days post-infection [42]. Aerosol exposure of macaques results in transient fever and viremia along with mild clinical signs such as loss of appetite, lethargy, hunched posture, and reduced movement [61,64,65]. However, macaques inoculated via aerosol with enzootic strains of VEEV develop a clinically relevant biphasic fever, as well as neurologic signs including ataxia, fever, depression, tremors, and hypothermia [52,60]. The disease progression in the macaque model is clinically relevant; infections feature low lethality and rare instances of neurologic sequelae. However, despite being consistent with human infections, this pattern is not ideal when attempting to better characterize the neurologic aspects of disease from a vaccine and therapeutic development perspective.

As neurologic signs of disease in macaques have been unreliable, recent applications of advanced telemetry to monitor intracranial pressure, body temperature, and EEG measurements have contributed greatly to the understanding of neurologic disease [59]. Combined with daily clinical observations, results of this telemetric study demonstrated biphasic fever as well as increases in intracranial pressure, while changes in EEG measurements indicated reduced sleep during the second bout of fever [59]. Common neurologic signs included mild tremors, twitching, and photophobia; ataxia and nystagmus were observed, but at infrequent rates [59]. Other research has applied the use of implantable devices to monitor ECG activity, demonstrating alterations correlated with fever [43]. Further application of such advanced telemetry will undoubtedly further elucidate the progression and severity of VEEV neurologic signs and sequelae that are poorly understood due to the lack of clinical data. While application of these tools may often be cost prohibitive in some cases, it will lead to more predictive preclinical evaluation vaccines and therapeutics and contribute to detailed mechanistic studies.

### 2.3. Western Equine Encephalitis Virus (WEEV)

WEEV is found throughout Canada, northern South America, Central America, and the western and central United States [13,14,66]. In North America, human and equine cases of WEEV have not been reported since 1994, and the virus itself has not been reported from mosquito populations since 2008 [67,68]. This dramatic reduction in case numbers is unlikely to be due to changes in virulence, but is likely due to changes in exposure rates reflecting ecologic drivers of enzootic circulation among avian hosts [69,70].

The WEEV incubation time ranges from 5 to 10 days [13]. Similar to the other encephalitic alphaviruses, the majority of human cases present with mild symptoms, including malaise, headache, myalgia, chills, and fever [13,71]. More severe disease presents with neurologic signs including confusion, coma, weakness, drowsiness, and irritability [13]. Similarly to VEEV and EEEV, severity of disease appears to be age related, with up to one-third of infants developing major complications, in contrast to an estimated 13% of adults [13]. The case fatality rate is estimated around 3%, although cases presenting with neurologic signs have increased fatality rates up to 15% [13,66,72].

Neurologic sequelae following infection occur in up to 30% of cases [14]. Half of surviving infants and children suffer permanent brain damage and sequelae [66,72,73], including behavioral changes, tremors, convulsions, seizures, photophobia, confusion, intellectual disability, irritability, depression, anxiety, paranoia, emotional instability, and deficits in hearing and speech [14,73,74,75]. Two reports indicate respiratory problems, with one patient diagnosed with alveolar hypoventilation and another with sleep apnea [14,76,77]. These sequelae may be permanent, with follow-up evaluations still indicating persistence through seven years post-infection [14,75].

Historically, WEE case reports offer perhaps the most insights on neurologic sequelae following any alphavirus infection [14]. Additionally, with its high rate and wide variety of sequelae, animal modeling of WEEV may greatly contribute to the understanding of the pathogenesis and mechanisms of viral encephalitis in general. Currently, research indicates that WEEV entry to the brain after subcutaneous exposure may occur in areas lacking a blood–brain barrier, including the hypothalamus, pineal body, area postrema, and the anteroventral third ventricle region [33]. Therefore, animal models that accurately reproduce both acute disease and sequelae are essential for the development of vaccines and much needed therapeutics. Table 3 outlines the neurologic signs of clinical infection in comparison to current animal models.

Murine WEE lethality appears to be age and strain specific [78,84,85]. WEEV tends to be lethal in mice up to five weeks old, following either subcutaneous or intraperitoneal infection, with lethality becoming strain specific in mice older than five weeks [78,86]. BALB/c, NIH Swiss, CD-1, and C57BL/6 mice all succumb to intranasal infection with the CO92 strain and develop signs of encephalitis [78]. NIH Swiss mice also feature 100% lethality following intranasal infection with the McMillan and TBT235 strains [78]. Aerosol and intranasal exposure of BALB/c mice also results in 100% lethality [53,79]. Golden Syrian hamsters infected through various routes demonstrate strain-specific lethality as well as histopathological encephalitis [70,80,81]. One study suggested neurologic signs during acute disease, described as “fits” during which hamsters would succumb to infection [81]. WEEV is lethal in a strain-specific fashion in guinea pigs, with virulence corresponding to lethality [82].

Cynomolgus macaques infected with WEEV via the subcutaneous route do not develop measurable viremia or readily detected clinical signs of disease [42]. Aerosol exposure may result in lethal disease in a strain-dependent manner [83,87]. Additionally, these primates may develop clinical signs that include fever and signs of encephalitis, such as reduced activity, tremors, convulsions, reduced response to stimuli, and coma [83]. Rhesus macaques serve as models for the teratogenic effects of WEEV, with infection during gestation resulting in spontaneous abortion and high rates of microcephaly, hydrocephaly, and encephalitis in fetuses [88,89,90].

## 3. Conclusions

In summary, alphavirus infections may cause debilitating disease with sequelae, whose mechanisms are yet to be fully elucidated. New World alphaviruses, including EEEV, VEEV, and WEEV, induce myriad neurologic sequelae, including seizures, coma, behavioral changes, cognitive impairments, and convulsions, among others [14,19,26,28,44,45,50,73,74,75]. Despite the relatively low numbers of reported cases [21,22,44,48,67,68], the rates of these neurologic sequelae are high with a lasting impact due to the lack of treatments to mitigate neurologic disease as well as recovery from sequelae [14,19,24,27,49].

Despite the importance of these alphaviral sequelae, animal modeling has yet to fully elucidate their mechanisms; no model reliably reproduces all neurologic aspects of human alphaviral encephalitis. Small animal models including mice and hamsters are often inconsistent in their reproduction of neurologic signs and sequelae based on age, route of infection, and virus strain [32,33,34,36,39,51,52,53,54,55,56,78,79,81,84,85]. While investigators typically view any neurologic signs and sequelae developed by rodents as making them cost-effective and accessible, nonhuman primates remain the gold standard for preclinical evaluation of therapeutics and likely will be critical to mechanistic understanding of neurologic disease and sequelae. Unfortunately, non-human primate models are also inconsistent in the development of neurologic signs and sequelae in a virus strain- and infection route-specific manner [35,38,40,41,42,52,59,60,61,83,87]. Additionally, they are also adept as masking neurologic signs and sequelae when researchers are present, limiting the usefulness of clinical observations. However, out of all available animal models, the neurologic signs and sequelae demonstrated by the non-human primate models are likely to be the most clinically accurate. The application of remote technologies to avoid the artifactual effects of direct observation of non-human primates is ideal.

Arguably the greatest recent contribution to understanding the various sequelae of neurologic alphaviral disease is the addition of advanced telemetry to non-human primate models [23,38,41,43,59]. Such detailed and nearly continuous measurements provide valuable insights into the development of neurologic disease and sequelae. Without such telemetry, measurements are limited to daily observations, weights, and body temperatures. Application of telemetric methods to the other encephalitic diseases would provide crucial insight to the extent of neurologic disease. However, the use of telemetry and non-human primates is cost-prohibitive for the vast majority of experiments. The further development of small animal models using extensive behavioral testing, perhaps used in conjunction with neuroimaging, may be more accessible and significantly increase understanding of neurologic sequelae. Use of these models will significantly aid in the development of detailed mechanistic studies as well as preclinical testing of vaccines and therapeutics. Finally, the virus strain specificity observed in the past with encephalitis alphavirus model infections can be overcome with the use of epidemiologically relevant alphavirus strains derived from cDNA clones to eliminate the effect of cell culture passages on virulence [91,92].

## Figures and Tables

**Table 1:** Clinical and animal modeling of neurologic disease with eastern equine encephalitis virus (EEEV) infection.

Species		Neurologic Signs	Route of Infection	References
Human	Clinical	Signs: vomiting, seizures, diarrhea, fever, headache, coma, drowsiness, behavioral changes Sequelae: seizures, convulsions, altered mental status, somnolence, intellectual disability	Aerosol or subcutaneous	[12,14,24,25,26,27,28]
Mouse	BALB/c	Tremors, altered behavior, fixed gaze, inability to respond to stimuli, reduced motor control	Intranasal	[34]
	C57BL/6	Tremors	Subcutaneous	[39]
Hamster	Golden Syrian	Coma, stupor	Subcutaneous	[36]
Guinea Pig	Hartley	Encephalitis	Aerosol	[32]
Non-human Primate	Cynomolgus Macaques	Tremors, seizures, twitching, ataxia, head pressing, nystagmus	Aerosol	[38,41]
		Ataxia, tremors, lethargy, imbalance	Cutaneous	[42]
	Marmosets	Tremors, behavioral changes, imbalance	Aerosol	[35,40]

**Table 2:** Clinical and animal modeling of neurologic disease with Venezuelan equine encephalitis virus (VEEV) infection.

Species		Neurologic Signs	Route of Infection	References
Human	Clinical	Signs: drowsiness, coma, seizures, convulsions, depression, disorientation, paralysis, encephalitis Sequelae: paralysis, convulsions, somnolence, seizures, coma, confusion, epilepsy, intellectual disability, altered personality, mental incapacity, photophobia, hearing loss, anosmia, ageusia, depression, recurrent headaches, fatigue	Aerosol or subcutaneous	[14,19,44,45,50]
Mouse	BALB/c	Succumb prior to neurologic signs	Aerosol or subcutaneous	[52,53,54]
	CD-1	Hind limb paralysis	Subcutaneous	[33,51,55]
	CB17	Paralysis	Subcutaneous	[56]
	SCID	Severe aggression, ataxia, unresponsiveness to stimuli	Subcutaneous	[56]
Hamster	Golden Syrian	Succumb prior to neurologic signs	Aerosol or subcutaneous	[57,58]
Non-human Primate	Cynomolgus Macaques	Ataxia, fever, depression, tremors, twitching, photophobia, nystagmus, hypothermia	Aerosol	[52,59,60]
		No clinical or neurologic signs	Cutaneous	[42,61]

**Table 3:** Clinical and animal modeling of neurologic disease with western equine encephalitis virus (WEEV) infection. ^a^ strain specific.

Species		Neurologic Signs	Route of Infection	References
Human	Clinical	Signs: confusion, coma, weakness, drowsiness, irritability Sequelae: behavioral changes, tremors, convulsions, seizures, photophobia, confusion, intellectual disability, irritability, depression, anxiety, paranoia, emotional instability, deficits in hearing and speech, alveolar hypoventilation, sleep apnea	Aerosol or subcutaneous	[13,14,73,74,75,76,77]
Mouse	BALB/c	Unspecified signs of encephalitis ^a^	Intranasal (CO92 strain)	[78]
		Succumb prior to neurologic signs ^a^	Aerosol and intranasal	[53,79]
	NIH Swiss	Unspecified signs of encephalitis ^a^	Intranasal (CO92 strain)	[78]
		Succumb prior to neurologic signs ^a^	Intranasal (McMillan and TBT235 strains)	
	CD-1	Unspecified signs of encephalitis ^a^	Intranasal (CO92 strain)	[78]
	C57BL/6	Unspecified signs of encephalitis ^a^	Intranasal (CO92 strain)	[78]
Hamster	Golden Syrian	Succumb prior to neurologic signs ^a^	Intraperitoneal	[70,80]
		Ataxia and neurologic “fits”	Respiratory, intracranial, intraperitoneal, and intradermal	[81]
Guinea pig	unspecified	Succumb prior to neurologic signs ^a^	Intracranial or intraperitoneal	[82]
Non-human Primate	Cynomolgus Macaques	Reduced activity, tremors, convulsions, reduced response to stimuli, coma	Aerosol	[83]
		No clinical or neurologic signs	Subcutaneous	[42]

## Data Availability

No new data were created or analyzed in this study. Data sharing is not applicable to this article.

## References

[1] (2021). International Committee on Taxonomy of Viruses: ICTV. Genus: *Alphavirus*. https://ictv.global/report/chapter/togaviridae/togaviridae/alphavirus.

[2] Jose J., Snyder J.E., Kuhn R.J. (2009). A structural and functional perspective of *Alphavirus* replication and assembly. Future Microbiol..

[3] Holmes A.C., Basore K., Fremont D.H., Diamond M.S. (2020). A molecular understanding of alphavirus entry. PLoS Pathog..

[4] Chen R., Mukhopadhyay S., Merits A., Bolling B., Nasar F., Coffey L.L., Powers A., Weaver S.C., ICTVReport Consortium (2018). ICTV Virus Taxonomy Profile: *Togaviridae*. J. Gen. Virol..

[5] Powers A.M., Brault A.C., Shirako Y., Strauss E.G., Kang W., Strauss J.H., Weaver S.C. (2001). Evolutionary relationships and systematics of the alphaviruses. J. Virol..

[6] Forrester N.L., Palacios G., Tesh R.B., Savji N., Guzman H., Sherman M., Weaver S.C., Lipkin W.I. (2012). Genome-scale phylogeny of the *Alphavirus* genus suggests a marine origin. J. Virol..

[7] Azar S.R., Campos R.K., Bergren N.A., Camargos V.N., Rossi S.L. (2020). Epidemic Alphaviruses: Ecology, Emergence and Outbreaks. Microorganisms.

[8] Centers for Disease Control and Prevention (2022). Eastern Equine Encephalitis Virus. https://www.cdc.gov/easternequineencephalitis/index.html.

[9] Lwande O.W., Lutomiah J., Obanda V., Gakuya F., Mutisya J., Mulwa F., Michuki G., Chepkorir E., Fischer A., Venter M. (2013). Isolation of tick and mosquito-borne arboviruses from ticks sampled from livestock and wild animal hosts in Ijara District, Kenya. Vector Borne Zoonotic Dis..

[10] Brown C.R., Moore A.T., Young G.R., Padhi A., Komar N. (2009). Isolation of Buggy Creek virus (Togaviridae: *Alphavirus*) from field-collected eggs of *Oeciacus vicarius* (Hemiptera: *Cimicidae*). J. Med. Entomol..

[11] Centers for Disease Control and Prevention (2020). Mosquito Bite Prevention: How to Protect Against Mosquito Bites. https://www.cdc.gov/mosquitoes/pdfs/MosquitoBitePreventionUS_508.pdf.

[12] Centers for Disease Control and Prevention (2021). Eastern Equine Encephalitis: Symptoms, Diagnosis, & Treatment. https://www.cdc.gov/easternequineencephalitis/symptoms-diagnosis-treatment/index.html.

[13] Defense Centers for Public Health—Aberdeen Western Equine Encephalitis 2022. https://phc.amedd.army.mil/PHC%20Resource%20Library/WesternEquineEncephalitis_FS_18-045-0317.pdf.

[14] Ronca S.E., Dineley K.T., Paessler S. (2016). Neurological Sequelae Resulting from Encephalitic *Alphavirus* Infection. Front. Microbiol..

[15] McKinney R.W., Berge T.O., Sawyer W.D., Tigertt W.D., Crozier D. (1963). Use of an Attenuated Strain of Venezuelan Equine Encephalomyelitis Virus for Immunization in Man. Am. J. Trop. Med. Hyg..

[16] Alevizatos A.C., McKinney R.W., Feigin R.D., Jaeger R.F. (1967). Live, Attenuated Venezuelan Equine Encephalomyelitis Virus Vaccine: I. Clinical Effects in Man. Am. J. Trop. Med. Hyg..

[17] Casamassima A.C., Hess L.W., Marty A. (1987). TC-83 Venezuelan equine encephalitis vaccine exposure during pregnancy. Teratology.

[18] Centers for Disease Control and Prevention (2018). Emergency Preparedness and Response: Bioterrorism Agents/Diseases. https://emergency.cdc.gov/agent/agentlist-category.asp.

[19] Guzman-Teran C., Calderón-Rangel A., Rodriguez-Morales A., Mattar S. (2020). Venezuelan equine encephalitis virus: The problem is not over for tropical America. Ann. Clin. Microbiol. Antimicrob..

[20] Silva M.L.C., Auguste A.J., Terzian A.C., Vedovello D., Riet-Correa F., Macário V.M., Mourão M.P., Ullmann L.S., Araújo J.P., Weaver S.C. (2017). Isolation and Characterization of Madariaga Virus from a Horse in Paraiba State, Brazil. Transbound. Emerg. Dis..

[21] Centers for Disease Control and Prevention (2021). Eastern Equine Encephalitis Virus: Statistics & Maps. https://www.cdc.gov/easternequineencephalitis/statistics-maps/index.html.

[22] Goldfield M.A., Welsh J.N., Taylor B.F. (1968). The 1959 outbreak of Eastern encephalitis in New Jersey. 5. The inapparent infection: Disease ratio. Am. J. Epidemiol..

[23] Trefry J.C., Rossi F.D., Accardi M.V., Dorsey B.L., Sprague T.R., Wollen-Roberts S.E., Shamblin J.D., Kimmel A.E., Glass P.J., Miller L.J. (2021). The utilization of advance telemetry to investigate critical physiological parameters including electroencephalography in cynomolgus macaques following aerosol challenge with eastern equine encephalitis virus. PLoS Negl. Trop. Dis..

[24] Lindsey N.P., Staples J.E., Fischer M. (2018). Eastern equine encephalitis virus in the United States, 2003–2016. Am. J. Trop. Med. Hyg..

[25] Dexter E.P., Dexter D.D., Lindsay C.W., Reichard R.R., Lutwick L. (2021). Case of fatal Eastern equine encephalitis. IDCases.

[26] Silverman M.A., Misasi J., Smole S., Feldman H.A., Cohen A.B., Santagata S., McManus M., Ahmed A.A. (2013). Eastern equine encephalitis in children, Massachusetts and New Hampshire, USA, 1970–2010. Emerg. Infect. Dis..

[27] Williams J.A., Long S.Y., Zeng X., Kuehl K., Babka A.M., Davis N.M., Liu J., Trefry J.C., Daye S., Facemire P.R. (2022). Eastern equine encephalitis virus rapidly infects and disseminates in the brain and spinal cord of cynomolgus macaques following aerosol challenge. PLoS Negl. Trop. Dis..

[28] Carrera J.P., Forrester N., Wang E., Vittor A.Y., Haddow A.D., López-Vergès S., Abadía I., Castaño E., Sosa N., Báez C. (2013). Eastern equine encephalitis in Latin America. N. Engl. J. Med..

[29] Villari P., Spielman A., Komar N., McDowell M., Timperi R.J. (1995). The economic burden imposed by a residual case of Eastern encephalitis. Am. J. Trop. Med. Hyg..

[30] Misra U.K., Kalita J., Phadke R.V., Wadwekar V., Boruah D.K., Srivastava A., Maurya P.K., Bhattacharyya A. (2010). Usefulness of various MRI sequences in the diagnosis of viral encephalitis. Acta Trop..

[31] Tsuchiya K., Katase S., Yoshino A., Hachiya J. (1999). Diffusion-weighted MR imaging of encephalitis. AJR Am. J. Roentgenol..

[32] Roy C.J., Reed D.S., Wilhelmsen C.L., Hartings J., Norris S., Steele K.E. (2009). Pathogenesis of aerosolized Eastern Equine Encephalitis virus infection in guinea pigs. Virol. J..

[33] Phillips A.T., Rico A.B., Stauft C.B., Hammond S.L., Aboellail T.A., Tjalkens R.B., Olson K.E. (2016). Entry Sites of Venezuelan and Western Equine Encephalitis Viruses in the Mouse Central Nervous System following Peripheral Infection. J. Virol..

[34] Phelps A.L., O’Brien L.M., Eastaugh L.S., Davies C., Lever M.S., Ennis J., Zeitlin L., Nunez A., Ulaeto D.O. (2019). Aerosol infection of Balb/c mice with eastern equine encephalitis virus; susceptibility and lethality. Virol. J..

[35] Porter A.I., Erwin-Cohen R.A., Twenhafel N., Chance T., Yee S.B., Kern S.J., Norwood D., Hartman L.J., Parker M.D., Glass P.J. (2017). Characterization and pathogenesis of aerosolized eastern equine encephalitis in the common marmoset (*Callithrix jacchus*). Virol. J..

[36] Paessler S., Aguilar P., Anishchenko M., Wang H.Q., Aronson J., Campbell G., Cararra A.S., Weaver S.C. (2004). The hamster as an animal model for eastern equine encephalitis—And its use in studies of virus entrance into the brain. J. Infect. Dis..

[37] Honnold S.P., Mossel E.C., Bakken R.R., Lind C.M., Cohen J.W., Eccleston L.T., Spurgers K.B., Erwin-Cohen R., Glass P.J., Maheshwari R.K. (2015). Eastern equine encephalitis virus in mice II: Pathogenesis is dependent on route of exposure. Virol. J..

[38] Albe J.R., Ma H., Gilliland T.H., McMillen C.M., Gardner C.L., Boyles D.A., Cottle E.L., Dunn M.D., Lundy J.D., O’Malley K.J. (2021). Physiological and immunological changes in the brain associated with lethal eastern equine encephalitis virus in Macaques. PLoS Pathog..

[39] Vogel P., Kell W.M., Fritz D.L., Parker M.D., Schoepp R.J. (2005). Early events in the pathogenesis of eastern equine encephalitis virus in mice. Am. J. Pathol..

[40] Adams A.P., Aronson J.F., Tardif S.D., Patterson J.L., Brasky K.M., Geiger R., De La Garza M., Carrion Jr R., Weaver S.C. (2008). Common marmosets (*Callithrix jacchus*) as a nonhuman primate model to assess the virulence of eastern equine encephalitis virus strains. J. Virol..

[41] Ma H., Lundy J.D., Cottle E.L., O’Malley K.J., Trichel A.M., Klimstra W.B., Hartman A.L., Reed D.S., Teichert T. (2020). Applications of minimally invasive multimodal telemetry for continuous monitoring of brain function and intracranial pressure in macaques with acute viral encephalitis. PLoS ONE.

[42] Smith D.R., Schmaljohn C.S., Badger C., Ostrowski K., Zeng X., Grimes S.D., Rayner J.O. (2020). Comparative pathology study of Venezuelan, eastern, and western equine encephalitis viruses in non-human primates. Antivir. Res..

[43] Ma H., Lundy J.D., O’Malley K.J., Klimstra W.B., Hartman A.L., Reed D.S. (2019). Electrocardiography Abnormalities in macaques after Infection with Encephalitic Alphaviruses. Pathogens.

[44] Aguilar P.V., Estrada-Franco J.G., Navarro-Lopez R., Ferro C., Haddow A.D., Weaver S.C. (2011). Endemic Venezuelan equine encephalitis in the Americas: Hidden under the dengue umbrella. Future Virol..

[45] Bowen G.S., Fashinell T.R., Dean P.B., Gregg M.B. (1976). Clinical aspects of human Venezuelan equine encephalitis in Texas. Bull. Pan Am. Health Organ.

[46] Quiroz E., Aguilar P.V., Cisneros J., Tesh R.B., Weaver S.C. (2009). Venezuelan equine encephalitis in Panama: Fatal endemic disease and genetic diversity of etiologic viral strains. PLoS Negl. Trop. Dis..

[47] Casals J., Curnen E.C., Thomas L. (1943). Venezuelan Equine Encephalomyelitis in Man. J. Exp. Med..

[48] Forshey B.M., Guevara C., Laguna-Torres V.A., Cespedes M., Vargas J., Gianella A., Vallejo E., Madrid C., Aguayo N., Gotuzzo E. (2010). Arboviral etiologies of acute febrile illnesses in Western South America, 2000–2007. PLoS Negl. Trop. Dis..

[49] Paessler S., Weaver S.C. (2009). Vaccines for Venezuelan equine encephalitis. Vaccine.

[50] Rivas F., Diaz L.A., Cardenas V.M., Daza E., Bruzon L., Alcala A., Hoz O.D.L., Caceres F.M., Aristizabal G., Martinez J.W. (1997). Epidemic Venezuelan equine encephalitis in La Guajira, Colombia, 1995. J. Infect. Dis..

[51] Charles P.C., Walters E., Margolis F., Johnston R.E. (1995). Mechanism of neuroinvasion of Venezuelan equine encephalitis virus in the mouse. Virology.

[52] Dupuy L.C., Richards M.J., Ellefsen B., Chau L., Luxembourg A., Hannaman D., Livingston B.D., Schmaljohn C.S. (2011). A DNA vaccine for venezuelan equine encephalitis virus delivered by intramuscular electroporation elicits high levels of neutralizing antibodies in multiple animal models and provides protective immunity to mice and nonhuman primates. Clin. Vaccine Immunol..

[53] Henning L., Endt K., Steigerwald R., Anderson M., Volkmann A. (2020). A Monovalent and Trivalent MVA-Based Vaccine Completely Protects Mice Against Lethal Venezuelan, Western, and Eastern Equine Encephalitis Virus Aerosol Challenge. Front. Immunol..

[54] Tretyakova I., Tibbens A., Jokinen J.D., Johnson D.M., Lukashevich I.S., Pushko P. (2019). Novel DNA-launched Venezuelan equine encephalitis virus vaccine with rearranged genome. Vaccine.

[55] Sharma A., Bhattacharya B., Puri R.K., Maheshwari R.K. (2008). Venezuelan equine encephalitis virus infection causes modulation of inflammatory and immune response genes in mouse brain. BMC Genom..

[56] Charles P.C., Trgovcich J., Davis N.L., Johnston R.E. (2001). Immunopathogenesis and immune modulation of Venezuelan equine encephalitis virus-induced disease in the mouse. Virology.

[57] Jahrling P.B., Scherer F. (1973). Histopathology and distribution of viral antigens in hamsters infected with virulent and benign Venezuelan encephalitis viruses. Am. J. Pathol..

[58] Jahrling P.B., Stephenson E.H. (1984). Protective efficacies of live attenuated and formaldehyde-inactivated Venezuelan equine encephalitis virus vaccines against aerosol challenge in hamsters. J. Clin. Microbiol..

[59] Ma H., Albe J.R., Gilliland T., McMillen C.M., Gardner C.L., Boyles D.A., Cottle E.L., Dunn M.D., Lundy J.D., Salama N. (2022). Long-term persistence of viral RNA and inflammation in the CNS of macaques exposed to aerosolized Venezuelan equine encephalitis virus. PLoS Pathog..

[60] Reed D.S., Lind C.M., Sullivan L.J., Pratt W.D., Parker M.D. (2004). Aerosol infection of cynomolgus macaques with enzootic strains of venezuelan equine encephalitis viruses. J. Infect. Dis..

[61] Rossi S.L., Russell-Lodrigue K.E., Killeen S.Z., Wang E., Leal G., Bergren N.A., Vinet-Oliphant H., Weaver S.C., Roy C.J. (2015). IRES-Containing VEEV Vaccine Protects Cynomolgus Macaques from IE Venezuelan Equine Encephalitis Virus Aerosol Challenge. PLoS Negl. Trop. Dis..

[62] Greene I.P., Paessler S., Anishchenko M., Smith D.R., Brault A.C., Frolov I., Weaver S.C. (2005). Venezuelan equine encephalitis virus in the guinea pig model: Evidence for epizootic virulence determinants outside the E2 envelope glycoprotein gene. Am. J. Trop. Med. Hyg..

[63] Scherer W.F., Chin J. (1977). Responses of guinea pigs to infections with strains of Venezuelan encephalitis virus, and correlations with equine virulence. Am. J. Trop. Med. Hyg..

[64] Tretyakova I., Plante K.S., Rossi S.L., Lawrence W.S., Peel J.E., Gudjohnsen S., Wang E., Mirchandani D., Tibbens A., Lamichhane T.N. (2020). Venezuelan equine encephalitis vaccine with rearranged genome resists reversion and protects non-human primates from viremia after aerosol challenge. Vaccine.

[65] Burke C.W., Froude J.W., Rossi F., White C.E., Moyer C.L., Ennis J., Pitt M.L., Streatfield S., Jones R.M., Musiychuk K. (2019). Therapeutic monoclonal antibody treatment protects nonhuman primates from severe Venezuelan equine encephalitis virus disease after aerosol exposure. PLoS Pathog..

[66] Kumar B., Manuja A., Gulati B.R., Virmani N., Tripathi B.N. (2018). Zoonotic Viral Diseases of Equines and Their Impact on Human and Animal Health. Open Virol. J..

[67] Bergren N.A., Auguste A.J., Forrester N.L., Negi S.S., Braun W.A., Weaver S.C. (2014). Western equine encephalitis virus: Evolutionary analysis of a declining alphavirus based on complete genome sequences. J. Virol..

[68] Robb L.L., Auguste A.J., Forrester N.L., Negi S.S., Braun W.A., Weaver S.C. (2019). Continued Evidence of Decline in the Enzootic Activity of Western Equine Encephalitis Virus in Colorado. J. Med. Entomol..

[69] Forrester N.L., Kenney J.L., Deardorff E., Wang E., Weaver S.C. (2008). Western Equine Encephalitis submergence: Lack of evidence for a decline in virus virulence. Virology.

[70] Bergren N.A., Haller S., Rossi S.L., Seymour R.L., Huang J., Miller A.L., Bowen R.A., Hartman D.A., Brault A.C., Weaver S.C. (2020). “Submergence” of Western equine encephalitis virus: Evidence of positive selection argues against genetic drift and fitness reductions. PLoS Pathog..

[71] Simon L.V., Coffey R., Fischer M.A. (2022). Western Equine Encephalitis.

[72] Weaver S.C., Kang W., Shirako Y., Rumenapf T., Strauss E.G., Strauss J.H. (1997). Recombinational history and molecular evolution of western equine encephalomyelitis complex alphaviruses. J. Virol..

[73] Palmer R.J., Finley K.H. (1956). Sequelae of encephalitis; report of a study after the California epidemic. Calif. Med..

[74] Herzon H., Shelton J.T., Bruyn H.E. (1957). Sequelae of western equine and other arthropod-borne encephalitides. Neurology.

[75] Earnest M.P., Goolishian H.A., Calverley J.R., Hayes R.O., Hill H.R. (1971). Neurologic, intellectual, and psychologic sequelae following western encephalitis. A follow-up study of 35 cases. Neurology.

[76] Cohn J.E., Kuida H. (1962). Primary alveolar hypoventilation associated with Western equine encephalitis. Ann. Intern. Med..

[77] White D.P., Miller F., Erickson R.W. (1983). Sleep apnea and nocturnal hypoventilation after western equine encephalitis. Am. Rev. Respir. Dis..

[78] Atasheva S., Wang E., Adams A.P., Plante K.S., Ni S., Taylor K., Miller M.E., Frolov I., Weaver S.C. (2009). *Chimeric alphavirus* vaccine candidates protect mice from intranasal challenge with western equine encephalitis virus. Vaccine.

[79] Nagata L.P., Hu W.G., Parker M., Chau D., Rayner G.A., Schmaltz F.L., Wong J.P. (2006). Infectivity variation and genetic diversity among strains of Western equine encephalitis virus. J. Gen. Virol..

[80] Julander J.G., Siddharthan V., Blatt L.M., Schafer K., Sidwell R.W., Morrey J.D. (2007). Effect of exogenous interferon and an interferon inducer on western equine encephalitis virus disease in a hamster model. Virology.

[81] Zlotnik I., Peacock S., Grant D.P., Batter-Hatton D. (1972). The pathogenesis of western equine encephalitis virus (W.E.E.) in adult hamsters with special reference to the long and short term effects on the C.N.S. of the attenuated clone 15 variant. Br. J. Exp. Pathol..

[82] Bianchi T.I., Aviles G., Sabattini M. (1997). Biological characteristics of an enzootic subtype of western equine encephalomyelitis virus from Argentina. Acta Virol..

[83] Reed D.S., Larsen T., Sullivan L.J., Lind C.M., Lackemeyer M.G., Pratt W.D., Parker M.D. (2005). Aerosol exposure to western equine encephalitis virus causes fever and encephalitis in cynomolgus macaques. J. Infect. Dis..

[84] Aguilar M.J. (1970). Pathological changes in brain and other target organs of infant and weanling mice after infection with non-neuroadapted Western equine encephalitis virus. Infect. Immun..

[85] Bianchi T.I., Aviles G., Monath T.P., Sabattini M.S. (1993). Western equine encephalomyelitis: Virulence markers and their epidemiologic significance. Am. J. Trop. Med. Hyg..

[86] Hardy J.L., Presser S.B., Chiles R.E., Reeves W.C. (1997). Mouse and baby chicken virulence of enzootic strains of western equine encephalomyelitis virus from California. Am. J. Trop. Med. Hyg..

[87] Burke C.W., Erwin-Cohen R.A., Goodson A.I., Wilhelmsen C., Edmundson J.A., White C.E., Glass P.J. (2022). Efficacy of Western, Eastern, and Venezuelan Equine Encephalitis (WEVEE) Virus-Replicon Particle (VRP) Vaccine against WEEV in a Non-Human Primate Animal Model. Viruses.

[88] Moreland A.F., Gaskin J.M., Schimpff R.D., Woodard J.C., Olson G.A. (1979). Effects of influenza, mumps, and western equine encephalitis viruses on fetal rhesus monkeys (*Macaca mulatta*). Teratology.

[89] London W.T., Levitt N.H., Altshuler G., Curfman B.L., Kent S.G., Palmer A.E., Sever J.L., Houff S.A. (1982). Teratological effects of western equine encephalitis virus on the fetal nervous system of *Macaca mulatta*. Teratology.

[90] Moreland A.F., Schimpff R.D., Gaskin J.M. (1979). Fetal mortality and malformations associated with experimental infections of western equine encephalomyelitis vaccine virus in rhesus monkeys (*Macaca mulatta*). Teratology.

[91] Rusnak J.M., Glass P.J., Weaver S.C., Sabourin C.L., Glenn A.M., Klimstra W., Badorrek C.S., Nasar F., Ward L.A. (2019). Approach to Strain Selection and the Propagation of Viral Stocks for Venezuelan Equine Encephalitis Virus Vaccine Efficacy Testing under the Animal Rule. Viruses.

[92] Gardner C.L., Sun C., Dunn M.D., Gilliland Jr T.C., Trobaugh D.W., Terada Y., Reed D.S., Hartman A.L., Klimstra W.B. (2022). In Vitro and IN Vivo Phenotypes of Venezuelan, Eastern and Western Equine Encephalitis Viruses Derived from cDNA Clones of Human Isolates. Viruses.

